# Prevalence of in-hospital mortality among adult patients with diabetic ketoacidosis in Ethiopia: a systematic review and meta-analysis of observational studies

**DOI:** 10.3389/fcdhc.2025.1501167

**Published:** 2025-04-08

**Authors:** Zenaw Debasu Addisu, Desalegn Getnet Demsie, Dessale Abate Beyene, Chernet Tafere

**Affiliations:** ^1^ Department of Clinical Pharmacy, College of Medicine and Health Sciences, Bahir University, Bahir Dar, Amhara, Ethiopia; ^2^ Department of Pharmacology, College of Medicine and Health Sciences, Bahir Dar University, Bahir Dar, Amhara, Ethiopia; ^3^ Department of Pharmacy, College of Medicine and Health Sciences, Debre Birhan University, Debre Birhan, Amhara, Ethiopia; ^4^ Department of Pharmaceutics, College of Medicine and Health Sciences, Bahir Dar University, Bahir Dar, Amhara, Ethiopia

**Keywords:** diabetic ketoacidosis, in-hospital mortality, Ethiopia, prevalence, diabetes mellitus

## Abstract

**Background:**

Diabetic ketoacidosis (DKA) is one of the most common life-threatening acute metabolic complications of diabetes, typically associated with disability, mortality, and significant health costs for all societies. In Ethiopia, available studies on in-hospital mortality rates of people living with DKA have shown high variability. Therefore, this systematic review and meta-analysis aims to summarize and provide quantitative estimates of the prevalence of in-hospital mortality among adult people living with DKA treated in Ethiopian hospitals.

**Methodology:**

A systematic literature search was conducted using MEDLINE, Embase, Google Scholar, Web of Science, and Africa-specific databases. Data were extracted in a structured format prepared using Microsoft Excel. The extracted data were exported to R software Version 4.3.0 for analysis. The I^2^ test was used to check the heterogeneity between primary studies with a corresponding 95% confidence interval (CI). Based on the test result, a random-effects meta-analysis model was used to estimate Der Simonian and Laird’s pooled effect on in-hospital mortality.

**Result:**

The review included a total of 5 primary studies. The pooled prevalence of in-hospital mortality among people living with DKA who received treatment in Ethiopia hospitals was found to be 7% (95% CI: 1-12). Most of the included studies reported that nonadherence to insulin treatment followed by infection was the most common triggering factor for the development of DKA.

**Conclusion:**

The prevalence of in-hospital mortality among people living with DKA was found to be 7%. This figure is unacceptably high compared to other published reports. Nonadherence to insulin treatment or antidiabetic medication and infection were identified as precipitating factors for developing DKA. Therefore, measures must be taken to improve medication adherence and decrease in-hospital mortality by providing ongoing health education on medication usage, effective in-hospital management of hyperglycemia, and increased access to high-quality care.

**Systematic review registration:**

https://www.crd.york.ac.uk/prospero/, identifier CRD42023432594.

## 1 Background

Diabetes mellitus is among the five major metabolic diseases worldwide (1). According to the Global Burden of Diseases, Injuries, and Risk Factors Study (GBD), in 2019, it was the eighth leading cause of death and disability, affecting nearly 460 million people across all countries and age groups (2). The prevalence of diabetes mellitus has risen dramatically in recent decades. Furthermore, by 2050, the number of people with diabetes is projected to exceed 1.31 billion (3).

Diabetic ketoacidosis (DKA) is one of the most common life-threatening acute metabolic complications of diabetes, and it is typically associated with disability, decreased life expectancy, and enormous health costs for all societies (4).

The severity of DKA is classified as mild, moderate, or severe based on the level of metabolic acidosis, which includes blood pH, bicarbonate, ketones, and the presence of altered mental status (5). Fasting plasma glucose greater than 250mg/dL, arterial pH 7.3, serum bicarbonate less than or equal to 18mEq/L, anionic gap greater than 10, and urine dipstick ketone level +2 are the most commonly used DKA diagnostic criteria (5). The basic principles of DKA management include rapid restoration of adequate circulation and perfusion with isotonic intravenous fluids, gradual rehydration and replacement of depleted electrolytes, insulin to reverse ketosis and hyperglycemia, and regular monitoring of clinical signs and laboratory tests to detect and manage complications and comorbid precipitating events (6). DKA is the leading cause of death among children and adolescents with type 1 diabetes, accounting for 50% of all deaths in this age group of diabetic patients (6). In adults with DKA, the overall mortality rate is relatively low; however, it exceeds 5% in elderly patients and those with significant comorbid conditions (7, 8).

Although the DKA mortality rate has decreased significantly in developed nations over the last two decades, from 7.96% to 0.67%, it remains high in developing countries, where it contributes to 12% of in-hospital mortality (9). In Africa, DKA mortality is significantly high, with studies from Kenya, Tanzania, and Ghana reporting death rates ranging from 26 to 29% (10, 11). Additionally, the causes of mortality vary between developed and developing countries; for instance, in advanced economies such as Japan and the Netherlands, complications like cerebral edema, adult respiratory distress syndrome (ARDS), and comorbid conditions significantly increase the risk of death (12–14). In contrast, in developing countries like India, Pakistan, and Bangladesh, sepsis, shock, and renal failure are more common contributors to DKA-related mortality (12, 15). Despite significant improvements in the knowledge, epidemiology, and management of DKA in the developed world, progress has been slower in developing nations, such as those in Sub-Saharan Africa (11).

Despite the high number of people living with DKA treated in Ethiopian hospitals, the prevalence of in-hospital mortality has not been thoroughly investigated. The incidence of hospital mortality among DKA patients in Ethiopia varies widely, ranging from 0% to 15% (16, 17). This variation, combined with the lack of a systematic review and meta-analysis on the pooled prevalence of in-hospital mortality for DKA patients in Ethiopia, underscores the need for a comprehensive review. Therefore, this systematic review and meta-analysis aims to summarize and provide quantitative estimates of the prevalence of in-hospital mortality among adult people living with DKA treated in Ethiopian hospitals.

## 2 Methodology

### 2.1 Protocol and reporting

This systematic review and meta-analysis is registered in the International Prospective Register of Systematic Reviews PROSPERO (CRD42023432594) and was conducted based on the Preferred Reporting Item for Systematic Review and Meta-Analyses (PRISMA) guideline.

### 2.2 Data source and searching strategy

A search was conducted for published primary articles related to mortality among adult people living with DKA receiving treatment in hospitals in Ethiopia. A systematic literature search was conducted using MEDLINE, Embase, Google Scholar, Web of Science, and Africa-specific databases (AFROLIB, African Index Medicus, and African Journals Online). The systematic search was conducted using the following keywords and MESH terms: (Prevalence OR Incidence OR Extent OR Magnitude) AND (“Diabetic Ketoacidosis” [Mesh] OR “Diabetic Ketoacidosis/drug therapy” [Mesh] OR “Diabetic Ketoacidosis/mortality” [Mesh] OR “Diabetic Ketoacidosis/therapy” [Mesh] OR “Diabetic Ketoacidosis treatment outcome”) AND “Ethiopia). This systematic review and meta-analysis included all relevant literature published from July 2014 to July 2024, reporting on the prevalence of in-hospital mortality among adult people living with DKA.

### 2.3 Study selection and eligibility criteria

To avoid duplications, all of the identified studies were exported to the EndNote citation manager and then assessed for eligibility to be included in this systematic review and meta-analysis using a prepared Microsoft Excel assessment format.

#### 2.3.1 Inclusion criteria

- Observational studies (cross-sectional, case-control, and cohort studies) with original data reporting in-hospital mortality people living with DKA were considered eligible to be included in this review.
- Research articles conducted in Ethiopia were included in this review
- Research articles enrolled adult patients (>18 years) with a diagnosis of DKA who presented to the emergency department, inpatient wards, and outpatient clinic, were included.
- Research articles that reported in-hospital mortality among adult DKA patients with either type 1 or type 2 DM, or both, were included
- Literature written in English or with an additional English translation was included
- Published literatures available from July 2014 to July 2024, were included.

#### 2.3.2 Exclusion criteria

- Articles with insufficient information, such as incomplete patient demographics or missing critical details necessary for inclusion, as well as records with missing outcomes of interest, personal opinions, editorial reports, letters to the editor, and qualitative studies, were excluded. Moreover, interventional studies were excluded to avoid treatment-related biases and obtain unbiased prevalence estimates.

### 2.4 Data extraction

All studies obtained using search strategies were exported to Endnote version 8 software, and the duplicates were removed. Finally, all studies were exported to a Microsoft Excel spreadsheet. The titles and abstracts of studies retrieved and those from additional sources were screened to identify studies that satisfy the inclusion criteria. Then, the full text of potentially eligible studies was assessed. The first author, study region, publication year, study design, and sample size were included in the data extraction format. Three reviewers (Z.D.A, D.A.B, and D.G.D) independently assessed the qualities of the articles. Discrepancies were resolved through discussion.

### 2.5 Quality assessment and risk of bias

The quality assessment tool was used to evaluate the quality of selected original studies. The Newcastle-Ottawa quality assessment scale adapted for cross-sectional studies was used to assess the quality of each original study (18). This scale was used to evaluate the internal and external validity, risk of bias, and methodological quality of each original study included. The quality assessment tool is divided into three sections. The first section concentrated on each original study’s methodological quality, such as objectives, sample size, and sampling technique. This section was graded on a 5-star scale. The tool’s second section evaluates study comparability and assigns a star rating out of two. The third section of the tool evaluates the outcome measures and data analysis and assigns a star rating out of three. The review and meta-analysis included studies with a score of 5 or higher.

The articles’ quality was assessed by two authors (Z.D.A, D.A.B, and D.G.D). They used the Newcastle-Ottawa quality assessment scale adapted for cross-sectional studies to evaluate each of the included articles individually. The authors then compared the scores assigned to each study. If the authors’ scores differed, it was discussed and resolved through consensus.

### 2.6 Outcomes

The primary outcome of this systematic review and meta-analysis was the pooled prevalence of in-hospital mortality among adult people living with DKA receiving treatment in Ethiopian hospitals. In some studies, mortality was reported as the overall prevalence of in-hospital mortality among adult DKA patients with both types of DM, without distinguishing between the two groups. Therefore, studies that reported the prevalence of in-hospital mortality among adult DKA patients with either type 1 or type 2 DM, or both, were included. The prevalence of in-hospital mortality was obtained from direct reports in the primary studies. Additionally, we included in-hospital mortality rates from studies where mortality was measured as an explanatory variable for other outcomes among patients receiving treatment in Ethiopian hospitals. Furthermore, the secondary outcome of the present systematic review and meta-analysis was to identify precipitating factors contributing to the development of DKA.

### 2.7 Statistical analysis

The extracted data were imported from the Microsoft Excel data extraction format to R software Version 4.3.0 for analysis. We checked the heterogeneity of primary studies using the I^2^ test. Based on the test result, a random-effects meta-analysis model was used to estimate Der Simonian and Laird’s pooled effect of in-hospital mortality. In addition, subgroup analysis was performed based on screening tools used to measure in-hospital mortality to minimize the random variations between the point estimates of the primary studies. Potential publication bias had also been examined through visual assessment of the funnel plot using Egger’s test (19). A P-value of <0.05 represents statistically significant publication bias. We performed sensitivity analyses to investigate the impact of individual studies on the pooled results, to examine changes in the degree of heterogeneity, and to verify the robustness of the study conclusion.

## 3 Result

### 3.1 Search results and characteristics of the included studies

**Figure 1:**
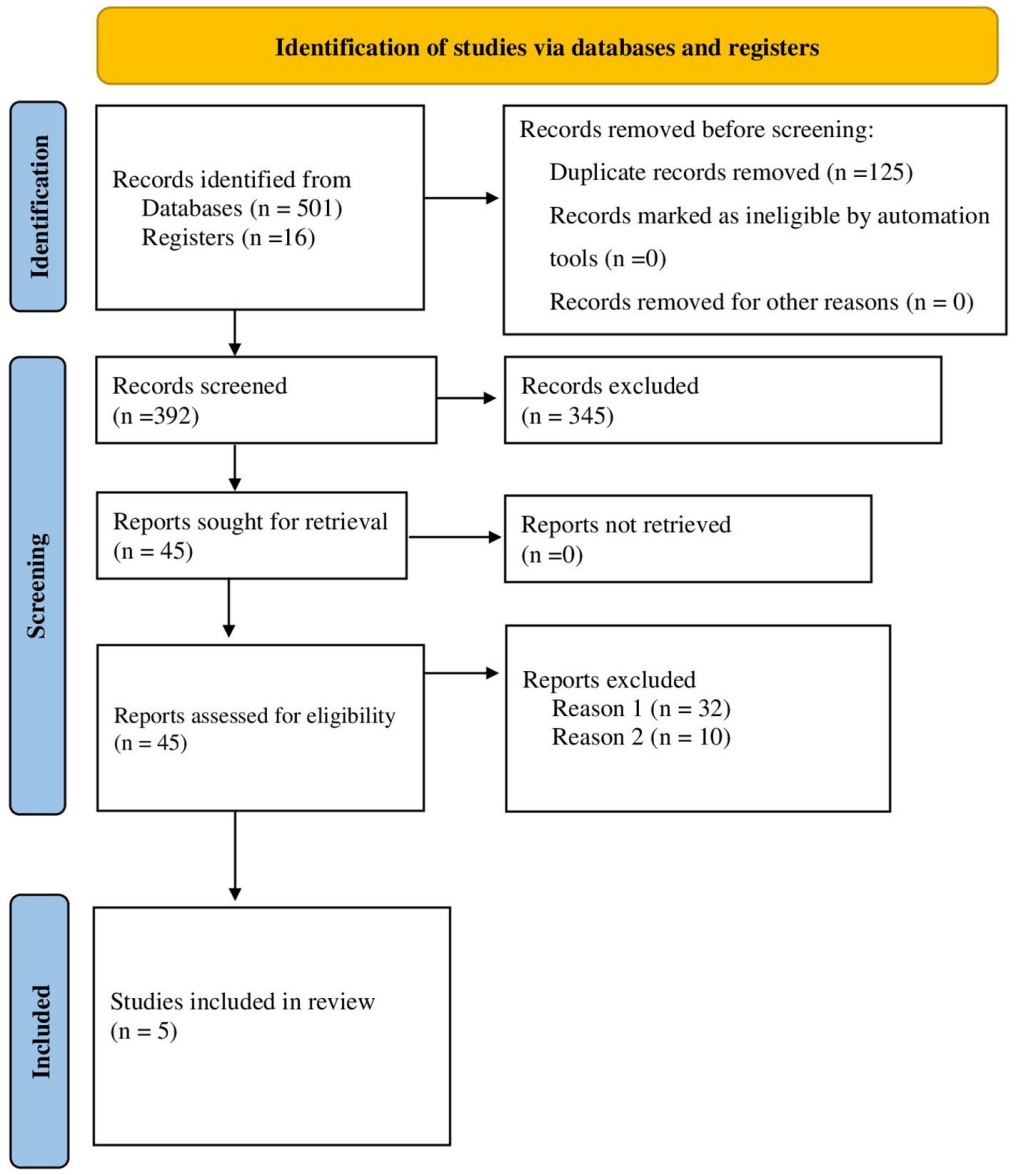
Flow diagram for study selection strategy as per PRISMA.

A total of 517 articles were obtained from the electronic database at primary search. Due to duplication, 125 articles were removed. The remaining 392 articles were evaluated by reading their title and abstracts. During title and abstract evaluation, 345 articles were excluded, and 47 articles were selected for further evaluation by reading their full texts. Following the full-text reading, 32 articles were excluded due to different primary endpoints of interest (Reason 1). Inappropriate results (Reason 2) led to the exclusion of 10 articles, while 5 articles met the inclusion criteria and were included in the systematic review and meta-analysis (**Figure 1**). These studies were conducted in different parts of Ethiopia 2 primary articles from Amhara region (20, 21) and,3 primary articles from Oromia region (16, 17, 22) were included. The sample size of the included primary studies was considerably variable ranging from 92 to 387 participants. Concerning age groups, all of the included studies included only adult patients (age >18 years). This systematic review and meta-analysis included a total of 1239 DKA patients. **Table 1** outlines the general characteristics of the articles.

**Table 1:** General characteristics of the articles: study design, sample size, mortality, and event rates.

Author, Publication year	Study region	Study design	Sample size	In hospital mortality	Event rate	Mortality rate %
Abegaz et al., 2018 (20)	Amhara	CS	387	17	0.044	4.4%
Debela et al., 2022 (16)	Oromia	RCS	178	0	0.0	0%
Kassaye et al., 2018 (22)	Oromia	CS	357	54	0.15	15%
Mezgebu et al., 2021 (21)	Amhara	CS	92	2	0.13	13%
Taye et al., 2021 (17)	Oromia	RCS	225	27	0.12	12%

Key; CS, Cross sectional; RCS, retrospective cross sectional; *Event Rate*, The proportion of people living with DKA who died during their hospital stay among the study population. *Mortality Rate (%)*, The percentage of people living with DKA who died during their hospital stay among the study population.

### 3.2 Quality assessment and score of included studies

Based on the Newcastle-Ottawa assessment scale, the quality score of the five studies assessed ranges from 7 to 10. **Table 2** shows the results of the scoring.

**Table 2:** Quality assessment of each study included in the review and meta-analysis based on the Newcastle-Ottawa assessment scale adapted for cross-sectional studies.

Author, Publication year	Methodological quality (5points)	Comparability of studies (2 points)	Outcome measures and analysis (3 points)	Total quality score (10 points)
Abegaz et al., 2018 (20)	5	2	3	10
Debela et al., 2022 (16)	3	2	2	7
Kassaye et al., 2018 (22)	5	1	2	8
Mezgebu et al., 2021 (21)	3	2	3	8
Taye et al., 2021 (17)	4	1	2	7

### 3.3 Outcome measures

**Figure 2:**
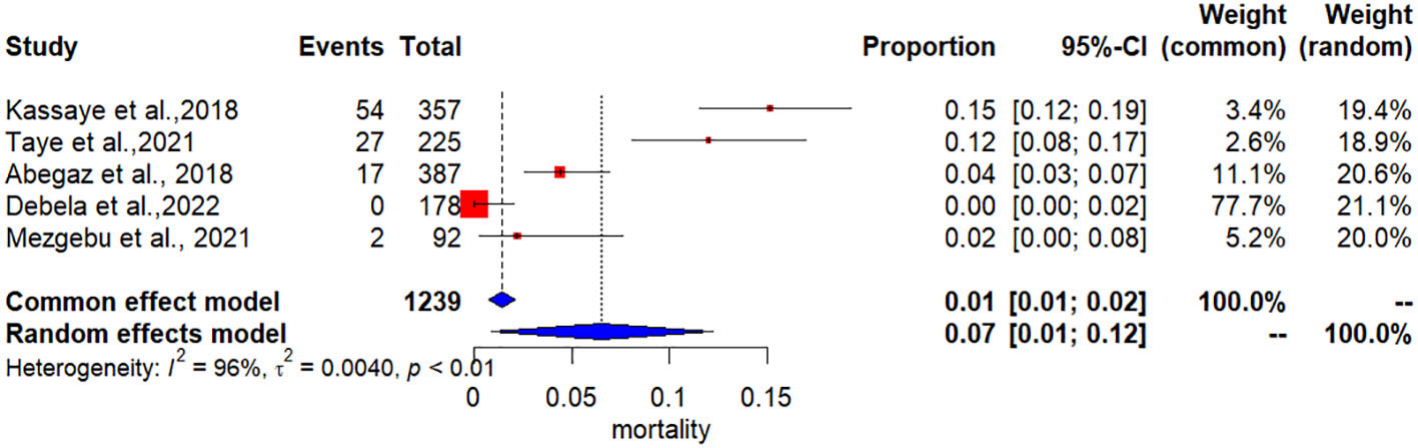
The pooled incidence of in-hospital mortality of Diabetic Ketoacidosis among patients receiving treatment in Ethiopian hospitals.

There was no in-hospital mortality from DKA, as reported by Debela et al., 2022 (16), while the highest incidence of in-hospital mortality from DKA, at 15%, was reported by Kassaye et al., 2018 (22). The pooled incidence of in-hospital mortality of DKA among patients receiving treatment in Ethiopian hospitals was 7% (95% CI: 1% to 12%). The pooled incidence of in-hospital mortality of DKA among patients receiving treatment in Ethiopian hospitals had significant heterogeneity (I^2^ = 96; P<.001) (**Figure 2**).

### 3.4 Subgroup analysis

**Figure 3:**
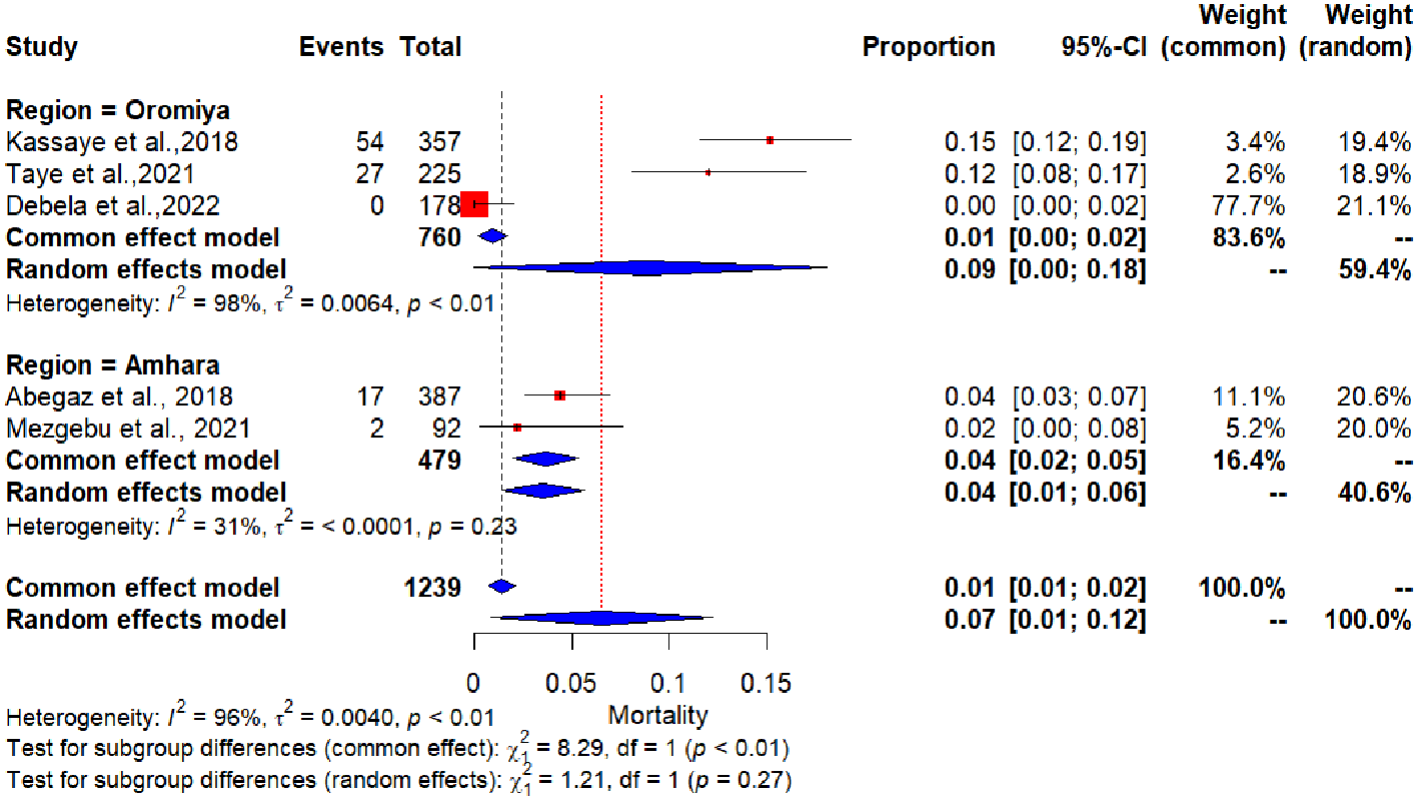
Forest plot showing subgroup analysis of the pooled incidence of in-hospital mortality of Diabetic Ketoacidosis by study regions.

A subgroup analysis of the pooled incidence of in-hospital mortality in DKA patients by study region revealed that the Amhara region had the lowest pooled proportion of poor treatment outcomes (40.6%), while the Oromia region had the highest (59.4%). However, the subgroup difference of random effect was not statistically significant (p = 0.27) (**Figure 3**).

### 3.5 Precipitating factor for the development of DKA

All articles included in this systematic review and meta-analysis reported precipitating factors for the development of DKA. Four of these studies (80%) reported a higher frequency of DKA participants triggered by nonadherence to insulin treatment or antidiabetic medication, followed by infection, which makes nonadherence the most common triggering factor for the development of DKA. One primary article reported that new-onset type 1 diabetes, followed by infections, was the most common precipitating factor for DKA as shown in **Table 3**.

**Table 3:** Precipitating factors for the development of DKA.

Author, Publication year	Precipitating factor	Frequency	Total
Abegaz et al., 2018 (20)	Infection	51 (13.17)	108
	Omission (nonadherence)	57 (14.71)	
Debela et al., 2022 (16)	Hypertension	1 (2.1)	46
	Infections	7 (14.71)	
	Missing Drug (Nonadherence)	9 (19)	
	More than one factors	5 (10.4)	
	No factors identified	24 (50)	
Kassaye et al., 2018 (22)	Infectious illness	248 (69.5)	357
	Nonadherence to insulin treatment	53 (14.80)	
	Other medical conditions	42 (11.80)	
	Others	14 (4.00)	
Mezgebu et al., 2021 (21)	Acute illness	4 (4.3)	96
	Drug discontinuation (non-adherence)	18 (40.9)	
	Infection	23 (25)	
	New-onset type 1 diabetes	46 (51)	
	Underdosing	5 (11.3)	
Taye et al., 2021 (17)	Infection	66 (29.3)	170
	Nonadherence to insulin treatment	91 (40.4)	
	Others	13 (5.8)	

Furthermore, three of the included studies reported the main reasons for non-adherence to insulin therapy or other antidiabetic medications. According to Kassaye et al., 2018 (22), the primary reason for discontinuing insulin was frustration with daily injections. Debela et al., 2022 (16) identified missed doses as the main reason for non-adherence, while Abegaz et al., 2018 (20) reported drug omission as the key factor.

### 3.6 Publication bias

**Figure 4:**
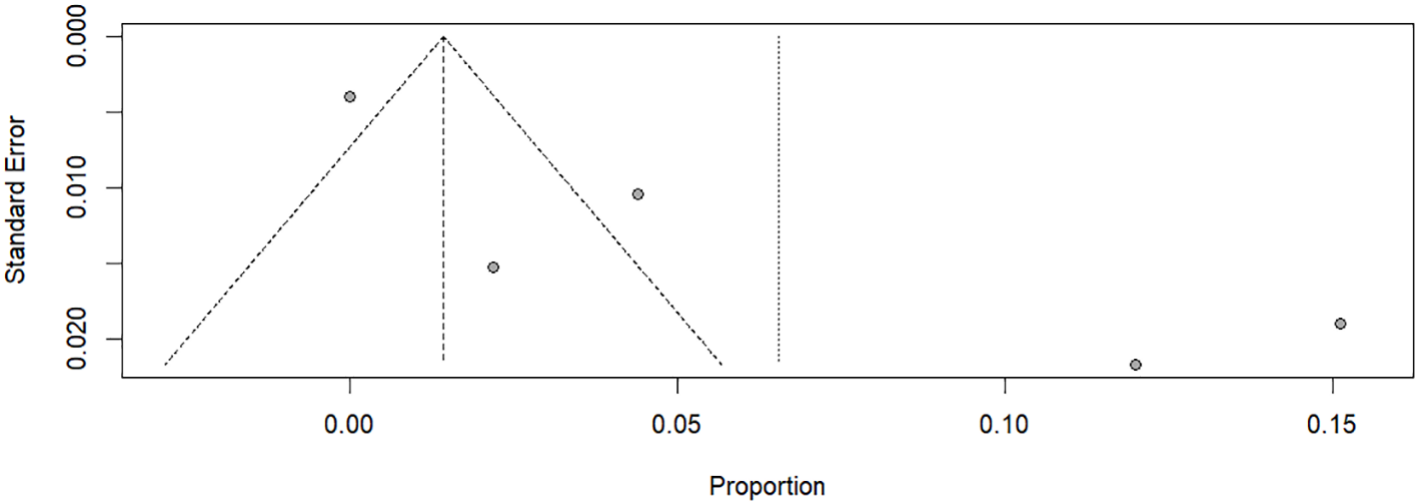
Funnel plot of the incidence of in-hospital mortality of Diabetic Ketoacidosis among patients receiving treatment in Ethiopian hospitals.

Visual inspection of Egger’s publication bias funnel plot, comparing standard error with the logit effect size, showed asymmetry. However, Egger’s test (P=0.21) did not indicate significant evidence of publication bias. Therefore, no publication bias was detected for the incidence of in-hospital mortality in DKA patients in Ethiopia (**Figure 4**).

### 3.7 Sensitivity analyses

The exclusion of studies with sample sizes less than 100 resulted in pooled incidences of 8% (95% CI: 1–14%, P < 0.001) for in-hospital mortality in DKA patients. The analysis showed no change in the degree of heterogeneity, and the pooled estimate of in-hospital mortality in DKA remained within the confidence interval of the pooled prevalence even when studies with small sample sizes were excluded. This indicates that studies with small sample sizes did not influence the overall result.

## 4 Discussion

The present systematic review and meta-analysis found that 7% of DKA patients in Ethiopian hospitals died while receiving hospital care. To the best of the authors’ knowledge, this is the first comprehensive review and meta-analysis conducted in Ethiopia to determine the precipitating factor for the development of DKA and the pooled incidence of hospital mortality in DKA patients.

The results of this systematic review and meta-analysis have a significant impact on improving the quality of care for hospitalized diabetic patients admitted with DKA by identifying common precipitating factors for the development of DKA and it also provides a summary of the hospital mortality rate and potential recommendations to improve outcomes. The review may also have clinical relevance and offer potential policy responses for Ethiopia’s healthcare systems. Hence, identifying common triggering factors is crucial for preventing the development of DKA besides to this, it improves the standard of care for DKA patients who are hospitalized and is essential to lowering hospital mortality.

The findings of the current systematic review showed that 7% (95 CI: 1% to 12%) of DKA patients in Ethiopian hospitals died while undergoing medical treatment. It is higher than a review done in developed countries, in treatment-experienced centers in, the Americas, and Europe, DKA mortality has decreased over time. The hospital mortality rate for DKA patients in these nations is currently reported as < 1% (23, 24). Moreover, the hospital mortality in Asia was reported to be 1.1% to 2.0% in Taiwan (25) and 3.3% in the Japanese study (26). The most probable reason for such a high mortality rate in Ethiopia could be due to lack of appropriate diabetic care, lack of screening for hypoglycemia and hypokalemia, financial constraints for laboratory support and insulin, as well as poor management practice of co-morbidity treatment like hypokalemia and hypoglycemia (24, 27, 28). Besides, it has been hypothesized that DKA mortality rates are highest where people cannot afford medical care (29). The current systematic review result is consistent with the analysis of data from West Africa, where the estimated in-hospital mortality rate was reported to be 7.9% (30). Numerous studies have revealed that, in most regions of Africa, DKA was the primary reason for death for those who needed insulin and were admitted to hospitals (31). Therefore, in developing nations like Ethiopia, in order to increase access to high-quality care, free medical treatment for patients admitted with DKA should be available.

Four of the studies included in this review reported a higher frequency of people live with DKA triggered by nonadherence to insulin treatment or antidiabetic medication, followed by infection.

The impact of poor treatment adherence on the occurrence of DKA has been highlighted in a number of recent research. Studies reported in India and Pakistan showed that insufficient insulin doses, and poor adherence to anti-diabetic treatment were the primary triggers of DKA (32, 33). Similarly, non-compliance to treatment regimens was the main contributing factor in the development of DKA in the USA among urban Afro-Caribbean communities and those who were uninsured (34). Poor treatment adherence to anti-diabetic treatment jeopardizes safety and treatment efficacy, increasing mortality and morbidity. As a result, the healthcare system will ultimately face high direct and indirect expenditures (35–37). Therefore, strategies to increase the accessibility of anti-diabetic medications, as well as health education on diabetic care and self-management, may help to increase diabetes patients’ adherence levels and may prevent the development of DKA. Besides, Infection was the most frequent DKA cause in the UK, followed by noncompliance (24). Hence, prevention and timely management of common infections among diabetic patients should be given great attention to hinder the development and mortality of DKA.

The subgroup analysis of the current systematic review and meta-analysis revealed that there was no significant difference between reports of pooled incidence of in-hospital mortality in DKA patients by study region.

This review and meta-analysis showed a high degree of heterogeneity among the included studies. Subgroup analysis based on the study region identified regional differences as a potential source of heterogeneity. In addition, variations in sample size and study quality scores may have contributed to this variability. Furthermore, differences in hospital infrastructure, resource availability, and diabetes care protocols across settings may have also played a role.

### 4.1 Strengths and limitations of the review

In this systematic review and meta-analysis, a thorough search of databases was carried out. A standardized measurement was used to assess the quality of the primary studies which were included in this review, and all of the articles complied with the requirements. However, the following issues can be considered to be the limitations of the current study. First, there were 5 studies with a high degree of heterogeneity. Furthermore, the included studies were from the Amhara and Oromia regions due to lack of data in other region, which may have an impact on the estimate of overall in-hospital mortality in DKA patients in Ethiopian hospitals who died while receiving medical care.

To improve the accuracy of prevalence estimates, we recommend conducting large-scale, nationwide multicenter studies across diverse regions of Ethiopia. This will provide a more comprehensive understanding of DKA mortality and inform targeted interventions to reduce its burden

## 5 Conclusion and recommendation

In this review and meta-analysis, the incidence of in-hospital mortality in DKA patients was found to be 7%. This figure is unacceptable high compared with other published reports. Therefore, measures must be taken to decrease in-hospital mortality by developing plans for effective in-hospital management of hyperglycemia along with increasing access to high-quality care for patients admitted with DKA recommended in Ethiopia.

## Data Availability

The original contributions presented in the study are included in the article/supplementary material. Further inquiries can be directed to the corresponding author.
